# DNA-directed coimmobilization of multiple enzymes on organic−inorganic hybrid DNA flowers

**DOI:** 10.3389/fbioe.2022.951394

**Published:** 2022-08-10

**Authors:** Yali Li, Jing Wang, Fenghong Huang, Yufei Zhang, Mingming Zheng

**Affiliations:** ^1^ Insititute of Food & Nutrition Science and Technology, Shandong Academy of Agricultural Sciences, Jinan, China; ^2^ Oil Crops Research Institute, Chinese Academy of Agricultural Sciences, Hubei Key Laboratory of Lipid Chemistry and Nutrition, Key Laboratory of Oilseeds Processing, Ministry of Agriculture, Wuhan, China

**Keywords:** DNA-directed immobilization (DDI), rolling circle amplification (RCA), multienzyme coimmobilization, organic-inorganic hydrid DNA flowers (hDFs), colorimetric detection of glucose

## Abstract

The artificial multienzyme systems developed by mimicking nature has attracted much interest. However, precisely controlled compositions and ratios of multienzymatic co-immobilization systems are still limited by the indistinguishable nature of enzymes. Herein, a strategy for fabricating DNA-directed immobilization of horseradish peroxidase (HRP) and glucose oxidase (GOx) on hybrid DNA nanoflowers (GOx-HRP@hDFs) is presented. The preparation of micron-sized hybrid DNA flowers (hDFs) begins with the predetermined repeatable polymer-like DNA sequences which contained two strands. The hDFs structure is generated through one-pot rolling circle amplification (RCA) and self-assembly with magnesium pyrophosphate inorganic crystals. Based on the rigid-base pairing, GOx and HRP conjugated with sequences complementary to strands would be anchored to the predesigned locations, respectively. By adjusting the loading amount/ratio of enzymes properly, the maximal catalytic efficiency can be precisely regulated. The reaction activity of GOx-HRP@hDFs was 7.4 times higher than that of the free GOx-HRP under the optimal mole ratio (GOx/HRP 4:1). In addition, this multienzyme catalyst system exhibits excellent precision, specificity, reproducibility, and long-term storage stability when applied to real human blood samples. The preceding results validate that GOx-HRP@hDFs are promising candidates for personal diabetes detection.

## Introduction

In living organisms, cell activities are directed by numerous synthetic pathways to maintain a healthy metabolism. The extraordinary synthetic effectiveness is partly determined by the cascaded biocatalysis in confined and highly organized microenvironments (Spoelstra et al., 2018; Tsitkov and Hess, 2019), since the cooperative enzymes bridge the gap between single biotransformation and permit the sequential reactions efficiently and timely. Besides, many enzymes work together in a multistep reaction or cascade to handle the highly complex networks of chemical reactions for life activities, like protein synthesis, cell defense, and DNA replication (Agapakis et al., 2012). Artificial multienzyme systems by mimicking natural enzyme organization are on the verge of rapid growth, particularly in diagnostics (Kucherenko et al., 2020), biotransformation (Liu et al., 2019) and biomedical engineering (Li et al., 2019). To create delicate microenvironments for multienzyme catalysis, the co-immobilization strategy of multiple enzymes was developed (Arana-Peña et al., 2021; Jiang et al., 2021). Two or several enzymes immobilized in the same space are not only in favor of improved enzymatic properties such as enhanced stability, selectivity, and reusability, but also accelerates the reaction rates. Random co-immobilization (Bilal et al., 2021) and compartmentalized immobilization (Liu et al., 2020) have been exploited for multienzyme cascade catalysis, which are relatively straightforward protocols that allow for convenient recovery and promoted stability of immobilized enzyme. However, indistinguishable enzyme compositions and unsupported microenvironments in nanoparticles are still the biggest challenges in the co-immobilization of multi-enzymatic systems (Wu et al., 2011).

DNA, as physicochemical stable macrobiomolecules, could hybridize with their complementary sequences through rigid Watson-Crick base pairing. The unique properties of DNA, such as programmable sequences, convenient synthesis, easy modification, and high stability, made them the ideal candidates for controllable multi-enzyme immobilization at molecular levels (Mirkin et al., 1996; Wilner et al., 2009; Jia et al., 2015). Recently, Yang (Yang et al., 2017) has reported coimmobiling both glucose oxidase (GOx) and horseradish peroxidase (HRP) through DNA-directed immobilization (DDI). The magnetic nanoparticles were available for fixation of two kinds of DNA sequences, so that the enzymes conjugated with corresponding DNA sequences could be captured through base-pairing. However, these methods were constrained by the elaborate design and complicated manipulation for reaching a homogeneous layout.

Rolling circle amplification (RCA) provides a potential solution since these enzymatic amplification methods offer a simple, rapid, and cost-effective strategy to produce significant amounts of long DNA strands via DNA polymerases. RCA is an isothermal enzymatic reaction that produces long single-stranded DNA with tandem repeating sequences complementary to the circular template. The RCA consists of three steps: first, linear padlock probes’ 5 and 3′ termini appropriately hybridize with target sequences. Then, Exonuclease I and Exonuclease III are added to the mixture to remove the non-circularized padlock probes. Finally, the capture probes act as primers and are catalyzed by phi29 DNA polymerase at constant temperature (Mohsen and Kool, 2016; Sakhabutdinova et al., 2017; Li et al., 2020). The polymer-like DNA strands and magnesium pyrophosphate inorganic crystals provide a composite organic-inorganic hybrid DNA flowers (hDFs) structure with remarkable structural properties during the RCA processes. The flower-like organizing functional materials have been fabricated for a variety of biological applications, including enhanced biocatalytic activity (Cheon et al., 2019; Kim et al., 2019), visual and colorimetric biosensing platform (Yan et al., 2018), targeted drug delivery (Mei et al., 2015) and multiplexed cellular imaging (Hu et al., 2014). The hDFs have advantages of high stability, facile functionalization, simple design and preparation (Zhou et al., 2007), in which only two single-stranded DNAs (primer and circular template) are needed. Moreover, the particle sizes of the DFs can be flexibly changed from nano-scale to micron-scale by controlling the RCA reaction conditions, thus facilitating the improvement of enzyme entrapment efficiency with more binding sites (Kim et al., 2019). Nevertheless, the strategies for both programmable arrangements and protected immobilization of enzymes in quantity remains the critical hurdle for successful biodiagnosis and biotransformation. Bi’s group (Yan et al., 2021) developed a RCA-based one-pot method to prepare the micron-sized DFs, which achieve the co-encapsulation and spatial regulation of GOx and HRP. The cascade of GOx/HRP is regulated by the formation of a highly ordered and hydrogen bonded water environment in the cavity of DFs, leading to an enhanced cascade catalytic efficiency compared with that in a homogeneous solution. However, it is hard to settle these DNA strands precisely with an optimized stoichiometric ratio and spatial uniformity. The DNA strands in the hDFs were tandemly and uniformly distributed, so the fully addressable DNA-based nanostructures have great potential for DDI. This, on the other hand, encouraged us to develop different nucleic acids that were adopted for more controllable configurations through the specific and predictable recognition for fabricating a multienzyme system that allows control of the stoichiometric ratio of the enzymes. The accurate layout of enzymes requires adherence to a rigorous numerical ratio and sequence order. Development of simple and versatile strategies for anchoring multienzyme uniformly and stoichiometrically has proven an urgent and challenging task.

Herein, we report a new approach that co-immobilizes different kinds of enzymes onto the hDFs. Through the one-pot RCA process, the predetermined repeated DNA sequences containing both strand A and strand B were systematically synthesized. Based on rigid-base pairing, the enzymes conjugated with sequences complementary to A or B would be anchored to the predesigned locations, respectively. On the other hand, the sequences A and B were designed with equal length and therefore nondistinctive binding forces, which guaranteed the non-preferential incorporation of both enzymes and the concomitant optimal synergism between these enzymes. By changing the loading amount/ratio properly, the microscopic distances between enzymes could be precisely regulated for minimal reciprocal inhibition and maximal catalytic efficiency. Glucose oxidase (GOx) and horseradish peroxidase (HRP) were employed for glucose detection to prove the high catalytic performance of our multienzyme system. Given the wide variety of possible enzyme associations and the high efficiency of this strategy, it is believed that this cascaded multienzyme immobilization strategy based on programmable hDFs can be extended to a wide range of biomedical applications such as new medical therapies and the manufacture of new pharmaceuticals.

## Materials and methods

### Materials and reagents

GOx (glucose oxidase from Aspergillus niger, >100 U) and HRP (peroxidase from horseradish, >300 U) were purchased from Sigma-Aldrich (St. Louis, MO). All DNA molecules used in this study were synthesized by Sangon Biotech Co. Ltd. (Shanghai, China). The DNA sequences were listed in Table 1. Phi29 DNA Polymerase (10 U µl^−1^), reaction buffer for phi29 DNA Polymerase were purchased from Thermo Fisher Scientific (MA, United States). T4 DNA Ligase (4 U µl^−1^), buffer for T4 DNA Ligase with 10 mM ATP were purchased from New England Biolabs. Inc (Beijing, China). dNTP mixture (2.5 mM) was purchased from Sangon Biotech Co. Ltd. (Shanghai, China). Tris (2-carboxyethyl) phosphine (TCEP), sulfosuccinimidy l-4-(N-maleimido-methyl) cyclohexane-1-carboxylate (sulfo-SMCC), 2,2′-azinobis-(3-ethylbenzthiazoline-6-sulphonate) (ABTS), bovine serum albumin (BSA), 4′,6-diamidino-2-phenylindole (DAPI), fluorescein isothiocyanate (FITC), and rhodamine B isothiocyanate (RhoB) were purchased from Aladdin (Beijing, China). The agarose was obtained from SolaiBao Technology Co. Ltd. (Beijing, China). All the other chemicals and reagents were of analytical grade. All chemicals were used as received without further purification. The solutions were prepared using ultrapure water which was purified by a UPR series ultrapure water device.

**TABLE 1:** Oligonucleotide sequences used in this assay^a^ ^,^ ^b^.

Oligonucleotide	Sequence (5–3′)	Number of bases
Template	ACC​GAA​TTT​GTGACT​GTA​ACA​TGG​GTG​GCA​TTATA​CCC​TGT​AGA	44
Primer	CAC​AAA​TTC​GGT​TCT​ACA​GGG​TAT	24
T_A_	ACC​GAA​TTT​GTG​ACT​GTA​ACA​T	22
T_B_	GGG​TGG​CAT​TAT​ACC​CTG​TAG​A	22

a Underlined sequences represent complementary region of Primer.

b T_A_ and T_B_ are the first half and the second half of the template, respectively.

### Synthesis of organic-inorganic hybrid DNA flowers

Preparation of circular DNA template: with the help of T4 DNA ligase, the circular DNA template was prepared using the primer and 5′-phosphorylated padlock probe. Primer (50 μM, 2.4 μl) and template (50 μM, 1.2 μl) were mixed in 10 × T4 DNA Ligase with 10 mM ATP reaction buffer (2 μl) and sterilized ultrapure water (10.5 μl). The solution was incubated at 95°C for 5 min and reduced the temperature by 0.4°C every 30 s to 16°C. Subsequently, 0.3 μl T4 DNA Ligase was added into the mixture at 16°C for 10 h.

Preparation of hDFs: the hDFs were self-assembled *via* RCA process. A typical RCA reaction was performed in a solution: the circular DNA template (12.5 µl), dNTPs (10 mM, 10 μl), phi29 DNA polymerase, 10 × Reaction buffer for phi29 DNA polymerase (20 μl), and sterilized ultrapure water (156.7 μl). The mixture was incubated at 30°C for 16 h. Then, the hDFs were separated through centrifugation at 10,000 rpm for 15 min. The resulting hDFs were washed three times with ultrapure water, then re-dispersed in ultrapure water and stored at 4°C before use.

### Conjugation of enzymes with oligonucleotide

For enzyme conjugations, GOx (1 mg) and sulfo-SMCC (2 mg) were dissolved in buffer A (1 ml, 10 mM PBS, pH 7.4, 0.1 M NaCl), respectively. Sulfo-SMCC (100 μl, 2.0 mg ml^−1^) was added dropwise into the solution of GOx (500 μl, 1.0 mg ml^−1^). The mixture was then shaken for 2 h at 37°C. The resulting mixtures of T_A_ and GOx were purified 3 times with Amicon-3K and Amicon-10K, respectively, using buffer A as an eluent. Single-stranded DNA-conjugated enzymes were prepared according to a previously reported method with a little modification. (Yang et al., 2016; Yang et al., 2017). Aqueous solutions of TCEP (15 μl, 30 mM) were combined with T_A_ (5 μl, 100 μM) in buffer B (45 μl, 20 mM PBS, pH 8.0) and incubated at 37°C for 2 h. The purified T_A_ and sulfo-SMCC-activated GOx solutions were combined and reacted at 29°C for 24 h with gentle shaking. The resulting T_A_-GOx conjugates were purified three times with Amicon-10K using buffer A to remove excess T_A_. The resulting T_A_-GOx conjugate solution was diluted to 1 ml. The final T_A_-GOx conjugate concentration was 50 μg ml^−1^. And the T_B_-HRP conjugates were prepared using the same approach.

### Synthesis of multienzyme catalysts through DDI (GOx-HRP@hDFs)

GOx and HRP were immobilized on the hDFs through the DDI technique. The T_A_-GOx conjugates and T_B_-HRP conjugates were mixed in a specific proportion, diluted in buffer A (1 ml), and then incubated with hDFs (12.5 μg) at 37°C for 3 h. The resulting multienzyme catalyst (GOx-HRP@hDFs) was rinsed thoroughly with buffer A to remove unbonded enzyme-DNA conjugates and then stored at 4°C for further study.

### Fluorescence-labeled enzymes

The preparation of FITC-labeled GOx, RhoB-labeled HRP and DAPI-labeled hDFs were adapted from a previously reported procedure (Zhang et al., 2014). A solution of FITC (5 mg ml^−1^ in DMSO, 0.2 ml) was added dropwise to a carbonate buffer (50 mM, pH 9.0, 2.0 ml) containing GOx (5 mg ml^−1^). The mixture was stirred at 25°C for 4 h before an NH_4_Cl aqueous solution (50 mM, 2 ml) was added. Then the solution was dialyzed against PBS (50 mM, pH 7.0) at 4°C for 48 h to remove the excess FITC. The RhoB-labeled HRP was prepared using the same approach. After enzyme of GOx-HRP@hDFs staining, DAPI was added into medium at 2% with a final concentration of 50 ng ml^−1^ and was incubated at 37°C for 1 h, then washed with H_2_O three times.

### Colorimetric detection of glucose

Glucose detection was performed as follows: Various amounts of glucose were dissolved in 1 ml the enzymolysis buffer (buffer A containing 0.5 mM ABTS) containing 2 mg GOx−HRP@hDFs nanocomposites, which was then incubated at 37°C. The nanocomposites from the above reaction were removed before the absorbance of the reaction product at 420 nm was recorded. The error bars in the figures were determined from the standard deviation of three measurements.

### Characterization

The surface morphologies of the samples were characterized by scanning electron microscopy (SEM; Zeiss Merlin Compact) with an energy-dispersive X-ray spectroscopy (EDX) detector and transmission electron microscopy (TEM; FEI Tecnai G2 F30). The immobilization of FITC-labeled GOx and RhoB-labeled HRP on the multifunctionalized DAPI-labeled hDFs was confirmed by confocal laser scanning microscopy (CLSM) on a Leica TCS SP5II CLSM system (Wetzlar, Germany). All the enzymatic assays were performed on a UV−vis spectrophotometer (UV-1900; Shimadzu, Japan) at room temperature. The Malvern Zetasizer Pro was used to measure zeta potentials.

## Results and discussion

**FIGURE 1:**
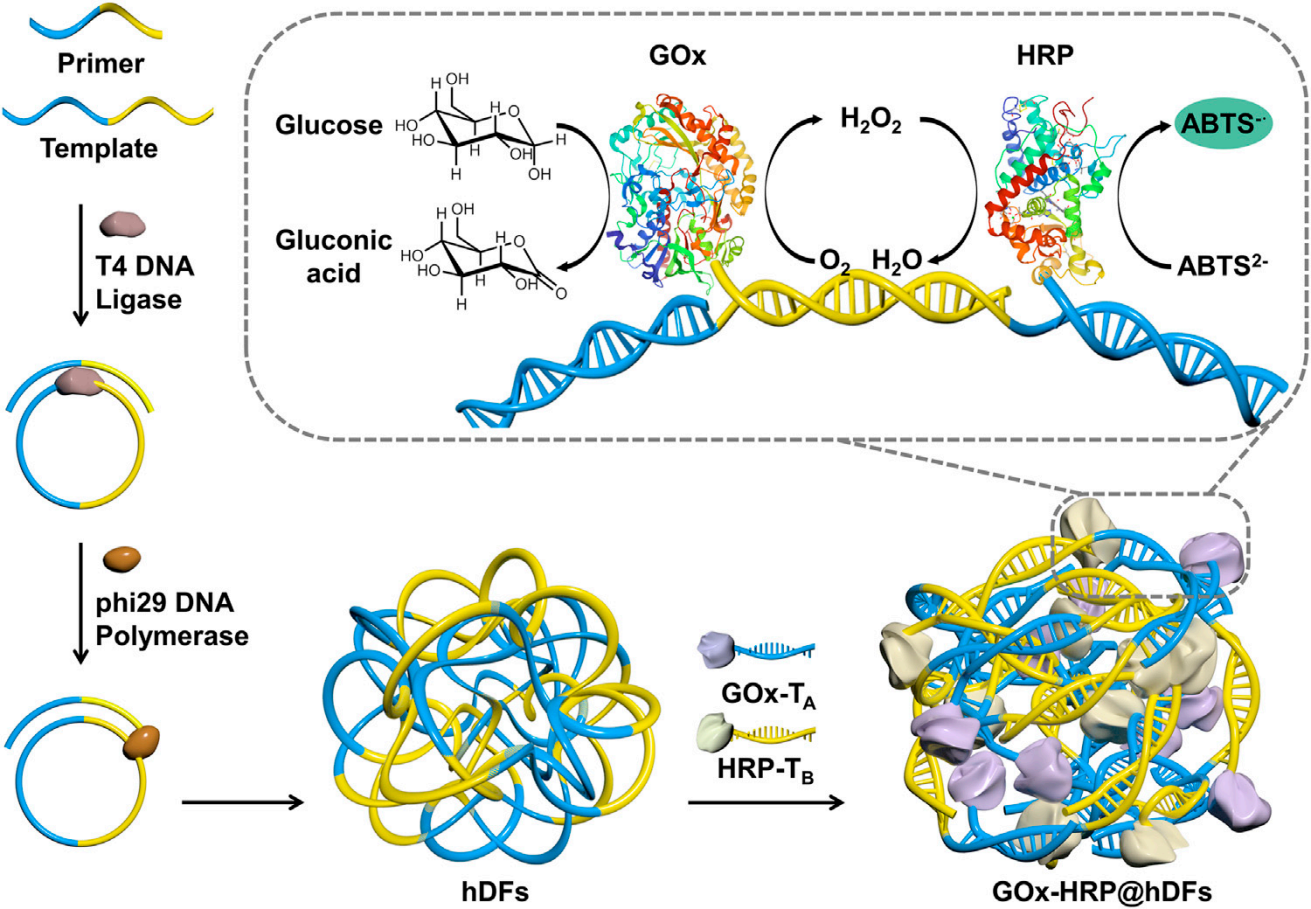
Schematic illustration of the preparation of hDFs through the one-pot RCA process, coimmobilized of GOx and HRP in hDFs via the DDI technique, and the mechanism for glucose detection using GOx/HRP@hDFs.

The strategy for the self-assembly process of GOx-HRP@hDFs via RCA and DDI is illustrated in Figure 1. Functional DNA moieties were simply incorporated into hDFs due to the sequence independence of hNFs assembly. The template should be a single-stranded circular nucleic acid or a single-stranded linear nucleic acid molecule. It promises that the template chain has a powerful enough targeting force as long as the base number of the primer is more than 6 bp. In this study, a 44 bp template chain and a 24 bp primer were designed. The primers can be complementarily paired with the two ends of the template chain (Table 1). Two ends of the padlock probe were engineered to be complementary to the primer. The padlock probe was hybridized with the primer after annealing and then circularized using T4 DNA ligase to generate a circular template. The addition of phi29 DNA polymerase and dNTPs which promoted the DNA production was due to an increased local DNA mass concentration and magnesium pyrophosphate, thus eventually leading to the formation of hDFs with flower-shaped and porous structures. The porous architectures of hDFs provided enough interaction sites for multienzyme immobilization. In this study, GOx and HRP were chosen as models. GOx catalyzes the oxidation of glucose to generate gluconic acid and H_2_O_2_ in the presence of dissolved O_2_. Then, the resulting H_2_O_2_ oxidized ABTS in the presence of HRP to green-colored oxidized ABTS, leading to the sensitive and selective detection of glucose. Sulfo-SMCC-activated GOx and HRP were conjugated with T_A_ and T_B_, respectively, (sequences complementary to A or B). (Niemeyer, 2002). GOx-T_A_ and HRP-T_B_ were coimmobilized to the predesigned locations on hDFs through DDI. Characterization of GOx-HRP@hDFs.

**FIGURE 2:**
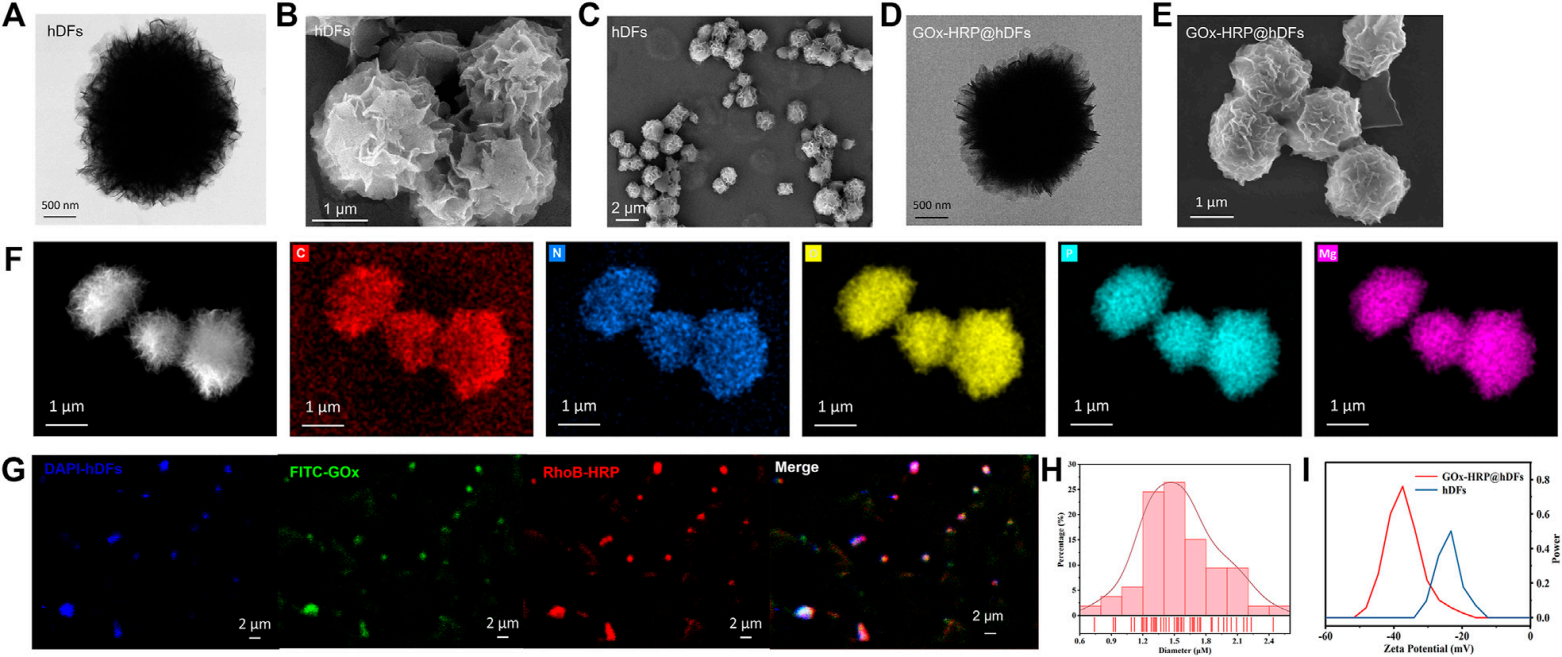
Morphology of the hDFs. **(A)** HAADF-STEM images of hDFs DNA hydrogel. **(B,C)** SEM images of hDFs. **(D)** HAADF-STEM images of GOx-HRP@hDFs. **(E)** SEM images of GOx−HRP@hDFs. **(F)** EDS elemental mapping of hDFs (bright field and elements: C, N, O, P, Mg in order). **(G)** Confocal laser scanning microscopy images of hDFs, on which GOx and HRP were simultaneously immobilized (GOx-HRP@hDFs). The GOx-HRP@hDFs, GOx and HRP were labeled with DAPI (blue), FITC(green) and RhoB (red), respectively. **(H)** Particle size distribution of hDFs. **(I)** Zeta potentials of hDFs and GOx-HRP@hDFs.

DNA-inorganic hybrid particles were fabricated based on an enzyme-driven synthetic strategy by DNA polymerase. During DNA polymerization, pyrophosphate anions are made as a byproduct. This drives the self-assembly of DNA-metal complex particles through a “two metal-ion” mechanism (Lee et al., 2017). The size distribution and morphologies of hDFs and GOx-HRP@hDFs were investigated by scanning electron microscopy (SEM) and transmission electron microscopy (TEM) (Figures 2A–E,H). The products consist of uniform and high-quality 3D flower-like hierarchical architectures of microspheres with fine monodispersity. As shown in Figure 2H, GOx-HRP@hDFs has a relatively narrow size distribution (65% of the particles’diameters ranging from 1.2 to 1.8 μm) and the average size is about 1.55 µm. The elemental composition of the products was examined by energy dispersion X-ray spectroscopy (EDX). As shown in Figure 2F, the EDX elemental mapping and spectra revealed that the presence of C, N, O, P and Mg in the hDFs. The confocal laser scanning microscopy (CLSM) images indicated the successful immobilization of GOx and HRP coimmobilization on the hDFs (Figure 2G). hDFs, GOx and HRP were labeled with DAPI, FITC and RhoB, respectively, and subjected to the same procedure to synthesize GOx-HRP@hDFs. From the microscopic view of CLSM images, a rather homogeneous distribution of luminescent blue particles generated from hDFs is clearly observed in the testing area. FITC-GOx (green) and RhoB-HRP (red) were anchored on the hDFs with the construction of spatial co-localization, which confirms the successful coimmobilization. Furthermore, the zeta potential of the hDFs and GOx-HRP@hDFs were characterized (Figure 2I). The potential of hDFs was measured to be −23.23 mV since the DNA is negatively charged. It has been reported that the isoelectric points of HRP and GOx are 7.2 and 4.2, respectively, (Wang et al., 2009). After loading negatively charged GOx and uncharged HRP under neutral conditions, the potential of GOx-HRP@hDFs was decreased to −37.43 mV.

### Optimization of the enzymatic activity of the multienzyme catalyst

**FIGURE 3:**
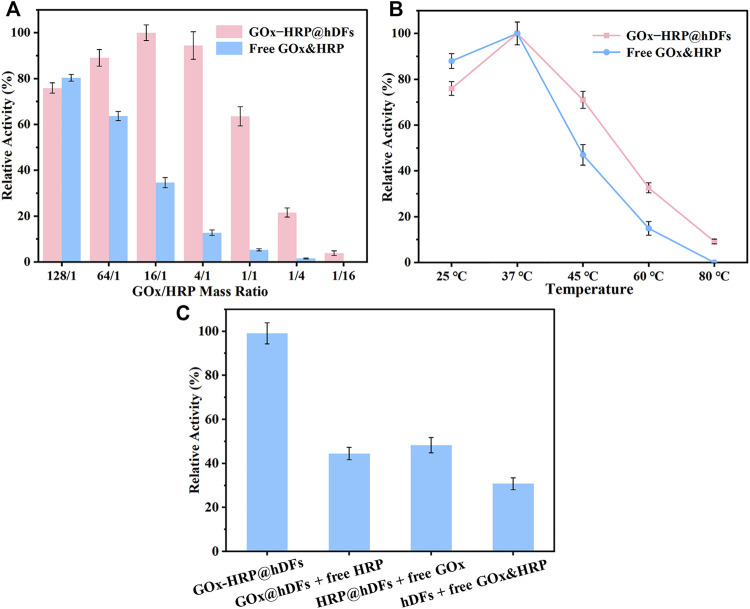
**(A)** Relative activity of the multienzyme system of GOx-HRP@hDFs and free GOx&HRP with different enzyme molar ratios. **(B)** Relative activity of free GOx&HRP and GOx-HRP@hDFs at different temperatures for 30 min. For each curve, the highest point was defined as 100% relative activity. **(C)** Relative activity of GOx-HRP@hDFs; free HRP and GOx@hDFs; free GOx and HRP@hDFs; and free HRP, free GOx and hDFs.

The relative stoichiometry of the multi-enzyme complexes in nature is optimized to maximize their catalytic efficiency depending on their different kinetic parameters. When the enzymes are in an unnatural system or hybrid mixture, they become spatially disorganized and lose their synergistic cooperation. It is an arduous task to achieve an optimal catalytic stoichiometry since the vast diversity in enzyme kinetics poses. Immobilized multienzyme systems can be designed by coimmobilizing suitable enzyme ratios in order to maximize overall productivity. To enhance the multienzyme cascade catalyst activity, the reaction conditions such as enzyme ratio and temperature have been investigated. The catalytic performance of GOx-HRP@hDFs and free GOx&HRP with different mass ratios of GOx and HRP were studied (Figure 3A). As the ratio of GOx and HRP of hDFs decreased from 128/1 to 16/1 (HRP remained the same), the reaction activity increased to the peak accordingly. As the ratio continued to decrease, the residual activity declined to only 3.85% compared with the top. The multienzyme cascade reaction activity had an inappreciable decrease compared with the best conditions of GOx-HRP@hDFs, while the GOx loading amount could be quartered with a ratio of 4/1. At 16/1 and 4/1, the relative enzymatic activity of GOx-HRP@hDFs was 2.9 and 7.4 times higher than that of the free GOx-HRP, respectively. Therefore, the mass ratio (4:1) was selected to prepare GOx-HRP@hDFs in subsequent experiments that achieved maximal overall activity with the minimum number of enzymes. Due to the favorable distance between cascade enzymes, they maintain higher local concentrations of intermediates and facilitate the diffusion from one enzyme to another. (Maffeis et al., 2021). Free GOx&HRP as controls with the same protein amount exhibited lower residual activity and showed a consistently decreasing trend with the ratio dropped. The activity reached its maximum value with the ratio closing to 128/1, which was better than the GOx-HRP@hDFs. The HRP random distribution reduced the collisions between enzyme and intermediates, lowering the enzyme’s catalytic activity. We then examined the temperature to improve the procedure. It is obvious that the optimum temperature for both systems was 37°C (Figure 3B). The activity of free GOx&HRP, GOx-HRP@hDFs increases at first and then drop down with the increase of temperature. Inappropriate temperature leads to enzyme denaturation and inactivation, resulting in a decrease of apparent enzyme activity. GOx-HRP@hDFs retained 32.6 and 9.2% activity even at 60 and 80°C, respectively. The DNA duplex provides important additional thermostability for the enzymes immobilized on hDFs. In contrast, the activity of free GOx&HRP declined dramatically and retained only 14.9% activity at 60°C, and no activity had been detected at 80°C. This result confirms the thermostability of GOx-HRP@hDFs systems. Furthermore, the activity of GOx-HRP@hDFs was compared to that of free HRP and GOx@hDFs; free GOx and HRP@hDFs; free HRP, free GOx and hDFs (Figure 3C). It was observed that the activity of GOx-HRP@hDFs was approximately 2.1–3.3 times higher than that of the other three bienzyme mixtures. The phenomenon is probably attributed to the high local concentrations of GOx and HRP trapped at support surfaces and higher effective concentration of intermediates (Sweetlove & Fernie, 2018). GOx-catalyzed H_2_O_2_ molecules can be enriched around the HRP, creating a substrate channeling environment that dramatically increases the activity of the cascade enzyme. (Cheon et al., 2019).

### Performance of colorimetric detection of glucose

**FIGURE 4:**
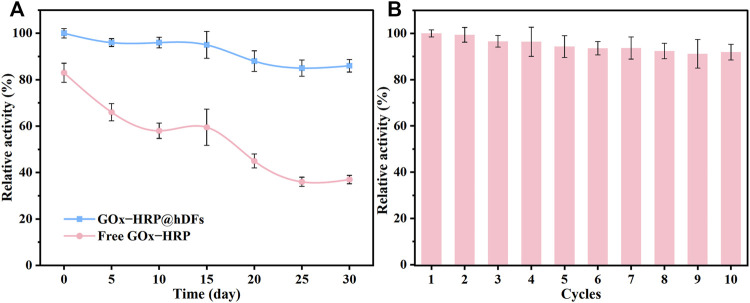
**(A)** Long-term stability of GOx-HRP@hDFs compared to free GOx&HRP incubated in PBS (0.01 M, pH 7.4) at room temperature. **(B)** Reusability of GOx-HRP@hDFs.

The GOx-HRP@hDFs was applied to the quantitative analysis of glucose under optimal conditions, and the results are shown in Figure 4. The maximum absorption peaks the in the UV-vis spectra increased gradually with the increase in glucose concentration (Figure 4A). Accordingly, it was clearly observed that the color of the solution gradually changed from colorless to dark green. The good linear relationship between the absorbance at 420 nm and glucose concentrations in the range of 0–20 μM, 20–150 μM with a low limit of detection (LOD) of 0.39 μM was achieved with color response (Figure 4C). LOD was calculated as 3 times the standard deviation of the blank sample divided by the regression slope, where the slope is derived from the calibration curve. GOx-HRP@hDFs used as a colorimetric glucose detector was comparable with or superior to some reported methodologies (Table 2).

**TABLE 2:** Comparison of GOx-HRP@hDFs with other peroxidase-mimicking nanozymes for colorimetric detection of glucose.

Carrier	Immobilization techniques	Linear range (μM)	Detection limit (μM)	Reference
a copper analogue of Prussian blue	encapsulation	-	0.48	Chen et al. (2022)
hierarchically porous metal-metalloporphyrin gel	encapsulation	0–350	0.5	Zhang et al. (2021)
magnetic Janus particles	DNA bridges	20–200	0.35	Shen et al. (2020)
hollow metal−organic frameworks	Protein-induced soft-templating pathway	0–1,500	0.4457	Liu et al. (2020)
enzyme−polymer	polymerization-induced co-assembly	0-1	0.13	Chiang et al. (2020)
		1.25–2.75		
metal−organic frameworks	DNA scaffold network encapsulation	1.10–140	0.4	Song et al. (2019)
poly-carboxybetaine	encapsulation	0.1–10	0.1	Zhu et al. (2020)
nanofiber-based strip	co-encapsulation	0.25–10	0.14	Luo et al. (2022)
cross-linked CS/PVA nanofiber	physical adsorption	2.7–13.8	2.7	Coşkuner Filiz et al. (2021)
MOFs and graphene oxide	single-stranded DNA with Janus properties	50–750	5.26	He et al. (2021)
multi-walled carbon nanotubes	supramolecular binding	2–410	0.31	Ortiz et al. (2019)
magnetic DNA nanocompartments	spatially confined	15.6–250	3.125	Song et al. (2020)
Fe_3_O_4_ magnetic nanoparticles	embed	5–150	2.5	Cheon et al. (2019)
alcell lignin and polylactic acid precursor blend	embed	150–2,700	89	Beaucamp et al. (2021)
hDFs	complementary base pairing	0.5–150	0.39	This work

To determine the specificity of the proposed strategy, the absorbance change at 420 nm was evaluated in the presence of common analytes using the optimized parameters. As shown in Figure 4B, barely fluorescence was generated when GOx-HRP@hDFs responded to the ions, such as KCl, NaCl, and CaCl_2_. Negative control saccharides including fructose, lactose, maltose, and mannose were also identified. Fructose and mannose showed minimal absorbance compared with glucose, even at tenfold higher concentrations. The selectivity of GOx-HRP@hDFs was further investigated by examining the effects of the potentially interfering substances commonly present in human blood, including glutathione, BSA and L-Ascorbic. Thus, there was no significant signal for the employed substances, indicating that the GOx-HRP@hDFs has high selectivity and anti-interference ability for glucose.

To further evaluate the applicability and reliability of GOx-HRP@hDFs in serum, the purchased samples of human serum were treated with diluted or additional glucose and then measured by the glucometer and colormetry. As shown in Table 3, The glucose levels were 1.36 and 4.05 mM for the original and diluted samples, respectively, which are close to the values of 1.4 and 4.2 mM obtained by glucometer. The values of recovered ratio for the assay of 2, 4 mM glucose added to the original serum samples was between 96.0 and 100.8%, while the 1, 2 mM glucose added in the diluted serum samples were between 94.1 and 94.6%. The recovery of various samples measured by the glucometer with different glucose concentrations was in the range of 106–115%. There was no significant difference between the glucometer and the method based on GOx-HRP@hDFs, which indicates that GOx-HRP@hDFs is applicable and reliable for detection of glucose in the real serum sample.

**TABLE 3:** Real sample analysis of the blood glucose levels in human serum based on a GOx-HRP@hDFs nanosystem.

Sample	Glucose added (mM)	Glucometer			GOx-HRP@hDFs		
		Found (mM)	RSD^b^ (%)	Recovery^c^ (%)	Found (mM)	RSD (%)	Recovery (%)
Original^a^	0	4.2	3.12	—	4.05	4.70	—
	2	6.6	1.98	106%	5.81	2.88	96.0%
	4	8.9	3.54	108%	8.12	1.07	100.8%
Diluted							
1/3	0	1.4	1.86	—	1.36	3.31	—
	1	2.7	2.19	113%	2.22	3.94	94.1%
	2	3.9	5.07	115%	3.18	2.85	94.6%

a Original amount of glucose in human blood serum.

b RSD is the relative standard deviation.

c Recovery = (measured glucose)/(original glucose + added glucose) × 100%.

### Stability and reusability

**FIGURE 5:**
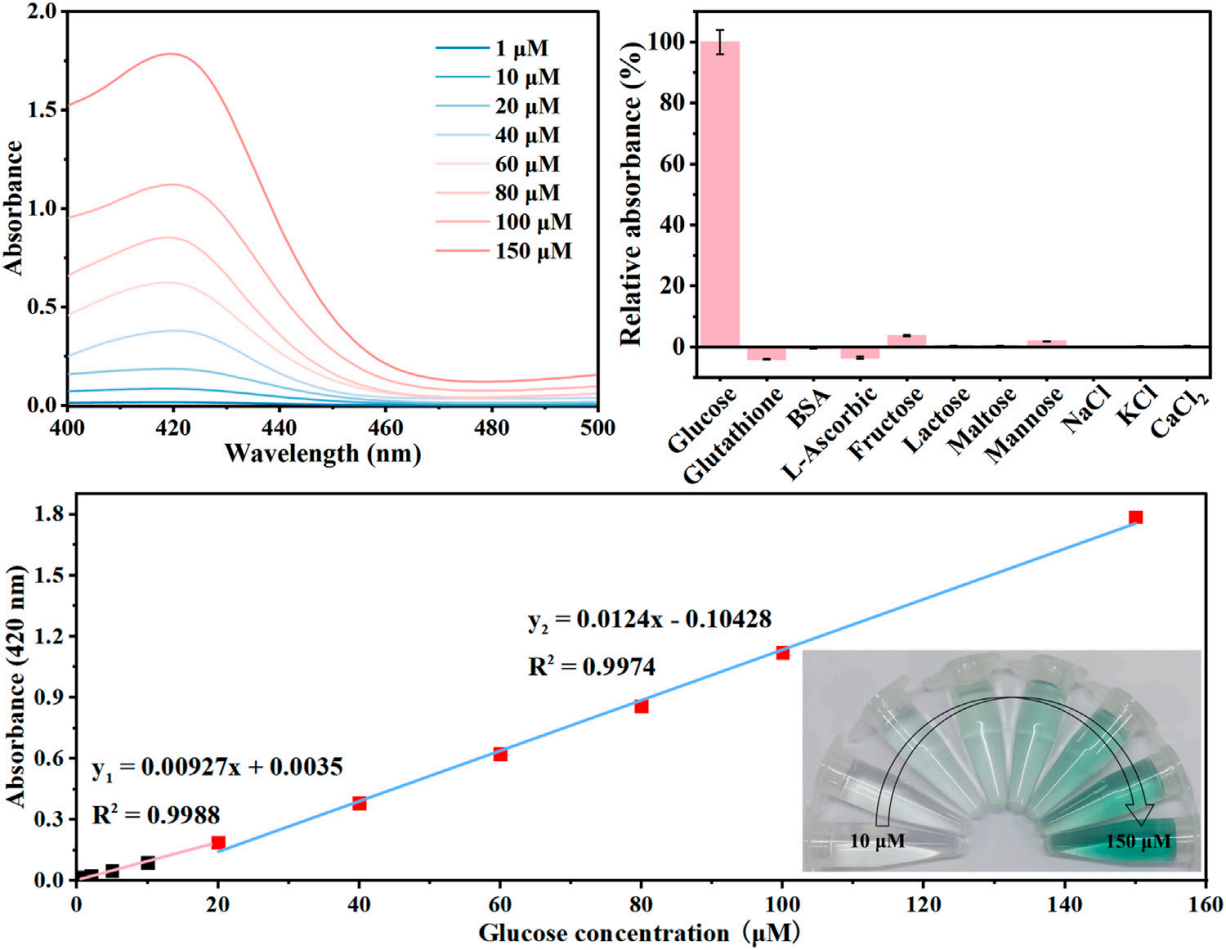
**(A)** UV-vis absorption data of solutions with the increase of glucose concentration from 1 to 150 μM; **(B)** selectivity and anti-interference ability of GOx-HRP@hDFs for 100 μM glucose in comparison to 1 mg/ml glutathione, BSA, L-ascorbic, 3 mM fructose, lactose, maltose, mannose, and NaCl, KCl, CaCl_2_. **(C)** plot of absorbance at 420 nm vs. the concentration of glucose. Inset: photographs showing visible detection of glucose in solution.

The temperature adaptability and long-term storage stability of GOx-HRP@hDFs were investigated. As shown in Figure 5A, the long-term storage stability of GOx-HRP@hDFs retained much higher activity compared to the free enzymes, which retained 86% of its initial activity even after 30 days, while the remained activity of free GOx&HRP dropped to 37.1%. The reusability of the immobilized enzyme is also a key factor for such a system. To study the reusability, GOx-HRP@hDFs was regained through centrifugation after the reaction. The reclaimed GOx-HRP@hDFs were reused in a fresh reaction solution. As shown in Figure 5B, GOx-HRP@hDFs retained a high degree of residual activity (more than 91%) after ten cycles, showing excellent reusability. Based on these results, it is concluded that co-immobilized enzymes onto the hDFs had much higher stability and activity compared to free GOx&HRP against the harsh environmental conditions, such as extreme temperatures and long-term storage.

## Conclusion

In summary, a strategy for fabricating multienzyme systems with GOx, HRP were developed with the organic-inorganic hybrid DNA flowers for sensitive detection of glucose level. The obtained GOx-HRP@hDFs precisely controlled the multiple enzymes coimmobilizing through DDI, which significantly enhanced the overall catalytic efficiency in comparison to the free enzyme. Therefore, this work provides a new route for the fabrication of artificial multienzyme systems. The difference absorbance at 420 nm showed a good linear relationship (*R* ^2^ = 0.999, 0.997) within a range of glucose levels from 0 to 20 μM, 20–150 μM with a pretty low LOD (0.39 μM). In addition, the GOx−HRP@hDFs showed excellent specificity to glucose, good temperature adaptability, and long-term storage stability even after 30 days of storage. The preceding results validate that GOx-HRP@hDFs are promising candidates for personal diabetes detection.

## Data Availability

The original contributions presented in the study are included in the article/Supplementary Materials, further inquiries can be directed to the corresponding authors.
